# Impact and Mechanisms of Action of BDNF on Neurological Disorders, Cancer, and Cardiovascular Diseases

**DOI:** 10.1111/cns.70138

**Published:** 2024-12-09

**Authors:** Min Lei, Qiwen Liu, Jinxia Nie, Rongyi Huang, Yan Mei, Dan Pan, Yong Chen, Wu Liu

**Affiliations:** ^1^ Hubei Key Laboratory of Diabetes and Angiopathy, Xianning Medical College Hubei University of Science and Technology Xianning Hubei China; ^2^ School of Basic Medical Sciences, Hubei Key Laboratory of Diabetes and Angiopathy, Xianning Medical College Hubei University of Science and Technology Xianning Hubei China; ^3^ Xianning Central Hospital The First Affiliated Hospital of Hubei University of Science and Technology Xianning Hubei China

**Keywords:** BDNF, cancer, cardiovascular diseases, neurogenic diseases, neuroregulation, TrkB

## Abstract

Brain‐derived neurotrophic factor (BDNF), which is primarily expressed in the brain and nervous tissues, is the most abundant neurotrophic factor in the adult brain. BDNF serves not only as a major neurotrophic signaling agent in the human body but also as a crucial neuromodulator. Widely distributed throughout the central nervous system (CNS), both BDNF and its receptors play a significant role in promoting neuronal survival and growth, thereby exerting neuroprotective effects. It is further considered as a guiding medium for the functionality and structural plasticity of the CNS. Increasingly, research has indicated the critical importance of BDNF in understanding human diseases. Activation of intracellular signaling pathways such as the mitogen‐activated protein kinase pathway, phosphatidylinositol 3‐kinase/protein kinase B/mammalian target of rapamycin pathway, and phospholipase C γ pathway by BDNF can all potentially enhance the growth, survival, proliferation, and migration of cancer cells, influencing cancer development. The loss of BDNF and its receptor, tropomyosin receptor kinase B, in signaling pathways is also associated with increased susceptibility to brain and heart diseases. Additionally, reduced BDNF levels in both the central and peripheral systems have been closely linked to various neurogenic diseases, including neuropathic pain and psychiatric disorders. As such, this review summarizes and analyzes the impact of BDNF on neurogenic diseases, cancer, and cardiovascular diseases. This study thereby aimed to elucidate its effects on these diseases to provide new insights and approaches for their treatment.

## Graphical abstract

Brain‐derived neurotrophic factor (BDNF) is not only the main neurotrophic signal in the human body but also an important neuromodulator. BDNF and its receptors are widely distributed throughout the central nervous system. They promote neuronal survival and growth and protect neurons. They are considered to be the guiding mediators of central nervous system functional and structural plasticity. Reduced BDNF levels in the central and peripheral systems are closely related to various neurological diseases, including neuropathic pain, inflammatory pain, and mental illness. More and more studies have shown that BDNF can potentially increase the growth, survival, proliferation, and migration of cancer cells, affecting cancer development. When BDNF and its receptor tropomyosin receptor kinase B are missing in the signaling pathway, brain and cardiovascular diseases are also more likely to occur. This article focuses on summarizing and analyzing the effects of BDNF on neurological diseases, cancer, and cardiovascular diseases to clarify its effects on these diseases and provide new ideas and methods for the treatment of these diseases.

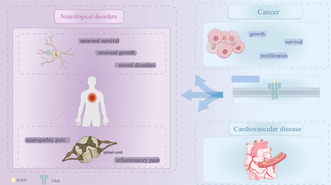

**Abbreviations**
β3AR: β3‐Adrenergic receptors
3′UTRs: 3′untranslated regions
Aβ: beta‐amyloid
ACS: acute coronary syndrome
AD: Alzheimer's disease
BDNF: brain‐derived neurotrophic factor
BDNF‐AS: BDNF Antisense RNA
cAMP: cyclic adenosine monophosphate
CCI: chronic constriction injury
CFA: Complete Freund's Adjuvant
CHD: coronary heart disease
CNS: central nervous system
CREB: cAMP‐response element binding protein
DRG: dorsal root ganglion
ERK: extracellular signal‐regulated kinases
GABA: gamma‐aminobutyric acid
GAD: GABA receptors
GluR: glutamate receptors
HIF1α: Hypoxic inducible factor1α
ICTF: 3,5,6‐trifluoro‐2′‐deoxyinosine
IP3: inositol 1, 4, 5‐triphosphate
JAK2: Janus kinase 2
JNK: c‐Jun N‐terminal kinase
KCC2: the electroneutral K + ‐Cl − cotransporters
LTP: long‐term potentiation
MAP‐1: MAPK signal transduction and activator of transcription1
MAPK: mitogen‐activated protein kinase
mTOR: mammalian target of rapamycin
NMDA: N‐methyl‐D‐aspartic
NT3: neurotrophic factors3
NT4: neurotrophic factors4
NTFs: neurotrophic factors
NTRK2: neurotrophic tyrosine kinase receptor
OSCC: oral squamous cell carcinoma
P2X4Rs: P2X4 purinergic receptors
P2X7 receptor: purinergicligand‐gatedionchannel 7 receptor
p75NTR: P75 neurotrophin receptor
PD: Parkinson disease
PI3K: phosphatidylin‐ositol‐3‐kinase
PLC‐γ: phospholipase Cγ
PSD: postsynaptic density protein
rACC: anterior cingulate cortex
RTK/RTKs: Receptor tyrosine kinase/receptor tyrosine kinases
SNI: spinal nerve injury
STAT3: Signal transducer and activator of transcription3
TrkB: tropomyosin receptor kinase B

## 1 Introduction

### 1.1 Background on Brain‐Derived Neurotrophic Factor

Neurotrophic factors (NTFs) are a special family of proteins that play a role in the growth and differentiation of both mature and immature neurons, serving as fundamental NTFs influencing central nervous system (CNS) function. In 1982, researchers extracted and purified a substance termed brain‐derived neurotrophic factor (BDNF) from the pig brain, which was shown to affect the survival of the nerve root ganglia [1]. BDNF has since been identified as one of the most widely distributed and studied NTFs in the mammalian brain. The functions of BDNF include the regulation of the development of neurons and glial cells, neuroprotection, and modulation of the short‐ and long‐term synaptic interactions crucial for cognition and memory [2]. From an evolutionary perspective, the BDNF gene sequence is highly conserved in mammals [3]. For example, the human BDNF gene has two distinct 3′untranslated regions, a feature that allows BDNF mRNA to be localized to different cellular regions during mRNA processing [4]. Another characteristic is that the BDNF gene is composed of nine exons (I‐IX, VH, and VIIIH) and has multiple splice sites and alternative promoters, resulting in the formation of various transcripts that encode proteins with different N‐terminal sequences and lengths during transcription [5]. These transcripts are sorted into different regions of neurons to exert their effects; for example, studies have observed a significant increase in the expression of BDNF exon IV in the hippocampus of rats that develop resilience to stress [6]. Other studies have shown that electroconvulsive therapy increases the expression of BDNF exons I and IV in the mouse cortex, thereby reversing dendritic spine atrophy induced by chronic stress [7]. Given the multiple possible transcripts of BDNF and their specific functions, different dendritic locations in hippocampal neurons can form synaptic connections with various neurons within the nervous system. Studies have shown that both patients with depression and animal models of depression exhibit neuronal dendritic atrophy and synaptic loss in the hippocampus [8]. As such, understanding how different BDNF transcripts are targeted to specific dendritic and synaptic sites within neurons could provide new insights into antidepressant research [9].

In addition to the aforementioned diseases, numerous studies have explored the association between BDNF and various other human diseases. Reduced BDNF expression is notably observed in psychiatric, neurodevelopmental, and neurodegenerative disorders [10]. Meanwhile, the expression of BDNF and its receptors has also been shown to be modulated in non‐neurological diseases, such as cancer and cardiovascular diseases, thereby influencing disease progression, with these effects being particularly significant in cancer. Evidence has indicated that BDNF expression levels are significantly elevated in cancer patients, while the modified forms of tropomyosin receptor kinase B (TrkB), which interacts with BDNF, are involved in various stages of cancer development, including the growth, maturation, migration, and invasion of cancer cells [11]. These findings have sparked significant interest in the use of BDNF as a diagnostic or prognostic biomarker of cancer [12] as well as in the development of drugs that target TrkB and its downstream pathways, such as first‐generation TrkB inhibitors [4]. In this review, we discuss the mechanisms through which BDNF functions in neurological diseases, cancer, and cardiovascular diseases.

### 1.2 Transmission of BDNF

**FIGURE 1:**
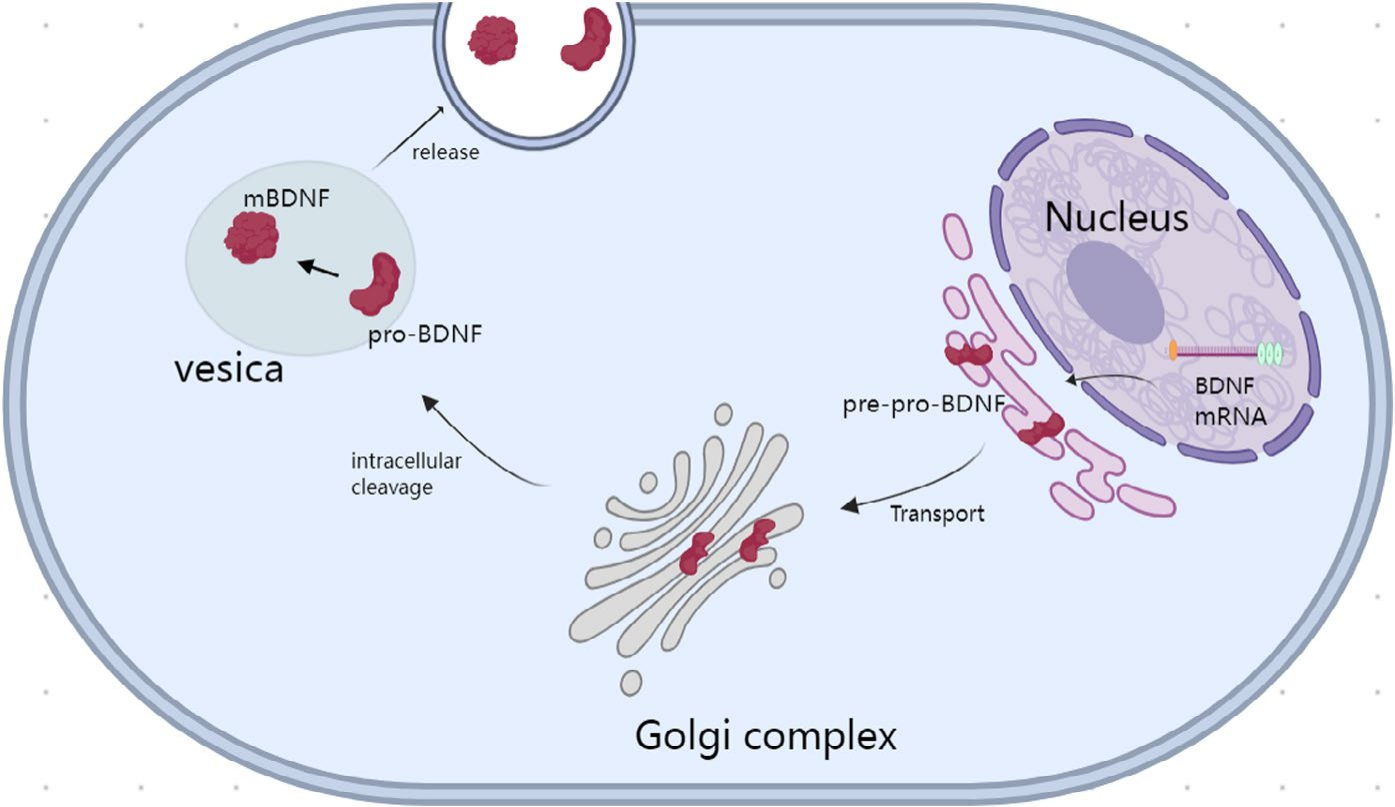
Synthesis and release of BDNF. Neurons can release mature BDNF, which is formed as a major nutritional signal or the neuroregulatory precursor pro‐BDNF, through transcription, translation, folding, cleavage, and packaging from presynaptic terminals. BDNF is first synthesized in a precursor form called pre‐pro‐BDNF, which is rapidly cleaved to form the precursor protein (pro‐BDNF). In intracellular pathways, the proBDNF precursor sequence is produced in the endoplasmic reticulum and transported to the Golgi apparatus. During intracellular cleavage, the pro‐region sequence is removed, leading to the formation of the immature neurotrophin precursor subtype (proBDNF) and the mature subtype of BDNF (m‐BDNF). The intracellular cleavage that forms m‐BDNF also occurs in intracellular vesicles and is transported to the axon terminal, from where it is subsequently released into the extracellular space through the presynaptic membrane.

BDNF is secreted by various cells, including neurons, and it has been shown to influence neuronal proliferation, differentiation, survival, and death. It can also cross the blood–brain barrier [13]. As mentioned earlier, BDNF can be translated into different proteins through the translation of different transcripts; however, these translated proteins are not initially functional but instead require further processing to become functional proteins, a characteristic similar to other NTFs [14]. BDNF is first synthesized in a precursor form called pre‐pro‐BDNF, which is rapidly cleaved to form the precursor protein (pro‐BDNF) [15]. Pro‐BDNF is subsequently cleaved again to generate mature BDNF (mature‐BDNF and mBDNF). Figure 1 presents an overview of the synthesis and release of BDNF. Notably, both pro‐BDNF and mBDNF aggregate into vesicles and are secreted extracellularly, exerting different functions [16]. Research has indicated that the balance between the two forms of BDNF plays a crucial regulatory role at different stages of brain development. Both pro‐BDNF and mBDNF can bind to the p75NTR receptor of the tumor necrosis factor receptor family, leading to various biological effects, including neuronal death, growth cone retraction, and the distinct pathways mediating axon degeneration during apoptosis and axon‐specific pruning [17]. However, pro‐BDNF has a stronger binding affinity for the p75NTR receptor than mBDNF. Additionally, studies have shown that mBDNF can act as a dimer on the TrkB receptor, activating multiple signaling pathways, including the phosphatidylinositol 3‐kinase (PI3K)/protein kinase B (Akt), NF‐κB, and mammalian target of rapamycin (mTOR) pathways [16] thereby regulating various biological activities of the nervous system, including neuronal survival, synaptic efficacy/plasticity, spine growth, and maturation. In a study on antidepressant treatments, it was elaborated that the transcription factor NF‐kB plays an important role in the pathophysiology of depression, participating in processes such as neurogenesis, synaptic transmission, and plasticity. BDNF plays a crucial role in most antidepressant treatments. Research suggests that the positive feedback loop between BDNF activation and the NF‐kB pathway may have potential beneficial effects in the antidepressant response.

The pro‐BDNF protein is encoded by the BDNF gene, which includes 11 exons and nine promoters. Several differences have been observed in the expression of the alternatively spliced human BDNF transcripts. In our previous study, we showed that anti‐BDNF transcripts were expressed in almost all the adult human tissues analyzed [5]. The C‐terminal of the precursor is sorted into the regulated secretory pathway after furin cleavage, whereas the amino‐terminus is either released constitutively, degraded, or sorted into a separate regulated secretory pathway. In cells with low furin levels, the precursor avoids cleavage, resulting in both sides of the precursor being sorted into the same regulated secretory vesicles [18]. Pro‐BDNF can be cleaved by both intracellular and extracellular proteases (such as plasmin and matrix metalloproteinases) to produce mature BDNF, which is approximately 14 kDa in size (mature‐BDNF and mBDNF), as well as several pro‐peptides. During cell division, BDNF and its propeptides are stored in dense presynaptic core vesicles in brain neurons [19]. Thus, neurons can release mature BDNF as a primary neurotrophic signal or neuromodulator. The precursor pro‐BDNF can further be synthesized and released from presynaptic terminals. However, BDNF can also be released in a retrograde manner from postsynaptic cells, thereby altering presynaptic activity [20] and mediating other specific functions in the developing cerebellum such as synapse elimination [21]. The mature form of BDNF primarily signals via TrkB, whereas pro‐BDNF binds to and activates sortilin and p75NTR. The latter is less expressed during development but is maintained at normal levels in adulthood [22]. Compared to pro‐BDNF, mBDNF can more effectively induce TrkB phosphorylation [23]. Regarding the balance between pro‐BDNF and mBDNF, studies on depression have shown that pro‐BDNF plays a detrimental role in mood regulation, whereas the overexpression of mBDNF can mitigate the damage caused by pro‐BDNF. This indicates that the balance between mBDNF and pro‐BDNF is crucial for maintaining brain homeostasis and preventing the development of depressive behavior. Therefore, when treating depression, restoring the balance between pro‐BDNF and mBDNF by inhibiting pro‐BDNF or supplementing mBDNF could be more effective than simply upregulating the BDNF gene [24]. Additionally, compared to pro‐BDNF, the effects of mBDNF primarily involve enhanced neuronal survival, growth, and synaptic activity [25]. The effects of mBDNF, which are mediated through the TrkB receptor, are highly diverse and can vary depending on multiple factors. New insights into the mechanisms that regulate and diversify BDNF biology in hippocampal neurons include the trafficking and subcellular compartmentalization of different BDNF mRNA species, the conversion of pro‐BDNF to mBDNF and BDNF pro‐peptide, the modulation of BDNF signaling by novel TrkB receptor interactors, and the pre‐ versus postsynaptic release of BDNF [26]. Furthermore, different molecular changes, including synaptic activity and plasticity, can be observed in the TrkB receptor between acute and slow activation, leading to downstream TrkB signaling [27]. Similarly, studies have shown that sustained TrkB activation promotes the growth of neuronal dendrites and the formation of spines [28]. Conversely, neuronal activity has been shown to promote TrkB surface enrichment. Research has further shown that after BDNF is secreted from active synapses and neurons, it recruits extrasynaptic TrkB into synapse‐rich membrane microdomains, leading to an increase in postsynaptic cyclic adenosine monophosphate (cAMP) concentration, which promotes the transport of TrkB receptors to the postsynaptic density (PSD). As such, the activity‐dependent regulation of TrkB receptor transport and surface expression depends on the extent of cellular and potentially synapse‐specific activity and BDNF release [29]. Moreover, studies have shown that glial cells, including astrocytes, can regulate BDNF recycling, while oligodendrocytes [30] and microglia are also known to be important targets and sources of BDNF. Finally, various TrkB receptor isoforms that mediate different functions have been identified. These findings indicate that endogenous BDNF is highly and strictly regulated in its synthesis, release, and signaling, allowing its diverse functions to be fully realized.

### 1.3 BDNF Receptor TrkB Signaling

**FIGURE 2:**
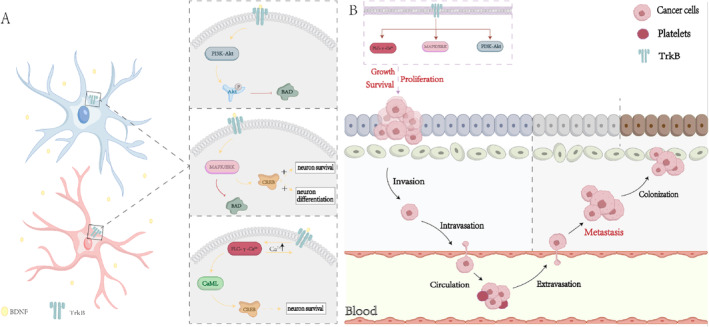
TrkB receptor signaling. A: Signal pathways related to the nervous system. BDNF and its receptor TrkB are widely distributed in multiple regions of the human brain. TrkB is a receptor tyrosine kinase. BDNF activates TrkB, exerting neuroprotective effects by inducing neurogenesis and synaptic plasticity. BDNF can bind to TrkB receptors and enhance synaptic plasticity while promoting neuronal protection by activating the MAPK/ERK, PI3K‐Akt, and PLC‐γ‐Ca^2+^ signaling pathways. The PI3K/Akt pathway regulates several proteins essential for neuronal survival. For example, the phosphorylation of Akt can inhibit the pro‐apoptotic function of the protein BAD, thereby exerting a protective effect on neurons. Simultaneously, the MAPK/ERK pathway can promote neuronal differentiation and survival by inhibiting the pro‐apoptotic protein BAD and activating the transcription factor CREB (cAMP response element‐binding protein, a key stimulus‐induced transcription factor). As for the PLC‐γ‐Ca^2+^ pathway, studies have shown that phospholipase C (PLC) associated with activated TrkB (via tyrosine 816 phosphorylation) may elevate intracellular Ca^2+^ levels and activate the calcium/calmodulin kinase pathway. This activation, in turn, activates CREB, promoting neuronal survival. B: Signal pathways related to cancer. Receptor tyrosine kinases (RTKs) are considered a class of receptors that play a crucial role in cancer progression. RTKs regulate various downstream signaling pathways, such as MAPK, PI3K/Akt, and PLC‐γ. The activation of these pathways can promote oncogenic processes, including cancer cell growth, proliferation, survival, migration, and epithelial‐mesenchymal transition (EMT).

BDNF and its receptor TrkB are widely distributed in multiple regions of the human brain. TrkB is a receptor tyrosine kinase (RTK) [31]. BDNF activates TrkB, exerting neuroprotective effects by inducing neurogenesis and synaptic plasticity [32]. BDNF can bind to TrkB receptors and enhance synaptic plasticity while promoting neuronal protection by activating the mitogen‐activated protein kinase (MAPK)/extracellular signal‐regulated kinase (ERK), PI3K‐Akt, and phospholipase C (PLC)‐γ‐Ca^2+^ signaling pathways [33]. The PI3K/Akt pathway regulates several proteins essential for neuronal survival. For example, the phosphorylation of Akt can inhibit the pro‐apoptotic function of the protein BAD, thereby exerting a protective effect on neurons [34]. Simultaneously, the MAPK/ERK pathway can promote neuronal differentiation and survival by inhibiting the pro‐apoptotic protein BAD and activating the transcription factor cAMP response element‐binding protein (cAMP, a key stimulus‐induced transcription factor) [35]. As for the PLC‐γ‐Ca^2+^ pathway, studies have shown that PLC associated with activated TrkB (via tyrosine 816 phosphorylation) may elevate intracellular Ca^2+^ levels and activate the calcium/calmodulin kinase pathway. This activation, in turn, activates cAMP‐response element‐binding protein (CREB), promoting neuronal survival [36]. RTKs are considered a class of receptors that play a crucial role in cancer progression. RTKs regulate various downstream signaling pathways, such as MAPK, PI3K/Akt, and PLC‐γ. The activation of these pathways can promote oncogenic processes, including cancer cell growth, proliferation, survival, migration, and epithelial‐mesenchymal transition (EMT) (Figure 2) [37]. For example, the activation of PI3K/Akt by BDNF/TrkB promotes the migration, anti‐apoptosis, and synthesis of pro‐survival proteins [38]. Previous studies have indicated that TrkB expression gradually increases in atypical hyperplasia and endometrial cancer. TrkB promotes EC cell growth, inhibits apoptosis, facilitates cancer cell migration, and enhances invasiveness. Thus, the overexpression of TrkB drives the EMT and changes in expression of molecular regulatory factors, including downregulation of E‐cadherin and upregulation of Twist. TrkB‐induced EMT has been associated with endometrial cancer [37]. Additionally, in Ishikawa cells, TrkB overexpression was shown to significantly increase the phosphorylation of Janus kinase 2 and signal transducer and activator of transcription 3, whereas TrkB knockout terminated their phosphorylation [39]. Recent studies have found that the upregulation of transcription factor differential gene 5 is associated with the promotion of EMT. Transcription factor differential gene 5 collaborates with lipoproteins to function as an extracellular signal sensor, promoting EMT in endometrial cancer [40]. Damage to the extracellular kinase signaling pathway of BDNF/TrkB can reverse the invasive and aggressive phenotypes caused by the upregulation of transcription factor differential gene 5 [41]. Furthermore, increased BDNF and TrkB signaling in neuroblastoma cells can act as a self‐secretion system promoting tumor growth, invasion, and metastasis. The BDNF/TrkB pathway may also be crucial in inducing angiogenesis in neuroblastoma [42]. Studies have shown that BDNF promotes vascular neogenesis by recruiting TrkB‐expressing active endothelial stem cells and promoting chemotherapy resistance in neuroblastoma cells [40]. Recently, the BDNF/TrkB pathway was also shown to activate the epidermal growth factor receptor, a widely upregulated growth factor receptor in many cancers. This activation was shown to be essential for the proliferation and migration of embryonic neurons, lung cancer cells, and ovarian cancer cells [37].

## 2 Impact and Mechanisms of Action of BDNF in Neurological Diseases

### 2.1 BDNF and Neuropathic Pain

Neuropathic pain is defined as pain caused by nervous system disorders. The conditions that may lead to neuropathic pain include diabetes, certain infections (such as shingles), nerve compression, nerve trauma, channelopathies, and autoimmune diseases. The development of animal models and new pharmacological strategies has sparked an interest in understanding the mechanisms underlying neuropathic pain. Neuropathic pain reflects the sensitization mechanisms in the peripheral and CNSs. Abnormal signals arise not only from damaged axons but also from intact nociceptors that share the innervation territory with damaged nerves [43].

In recent years, an increasing number of studies have shown that BDNF is not only related to tumor progression and nervous system development but is also closely associated with the occurrence of neuropathic pain [44]. Further, several studies have shown that BDNF plays a dual role in the regulation of neuropathic pain. For example, in the dorsal root ganglia (DRG) and spinal cord, BDNF induces pain development through P2X7 and N‐methyl‐d‐aspartate (NMDA) receptors [45]. However, BDNF has a neuroprotective effect and can alleviate pain in higher central structures, such as the hippocampus and cortex [46].

Moreover, most studies have indicated that BDNF primarily exerts an analgesic effect in the higher central regions, playing only a minor role in causing pain. For example, the epigenetic modifications of BDNF in the cerebral cortex can lead to neuropathic pain. Research on the function of the brain in neuropathic pain models is limited, thus providing an opportunity for researchers to explore this area and identify effective therapeutic targets for pathological pain [47].

#### 2.1.1 The Role of BDNF in the Peripheral Nervous System

In the peripheral nervous system, BDNF is synthesized by DRG neurons and transported retrogradely from the cell body to the terminal cells in the spinal cord [48]. In this section, we discuss the impact of BDNF on spinal nerves based on relevant research. Ding et al. previously validated the involvement of spinal BDNF in the long‐term excitability (central sensitization) of wide dynamic range neurons in the dorsal horn of rats undergoing spinal nerve ligation (SNL), as well as the development of abnormal pain, finding that BDNF induces the upregulation of GluN2B‐NMDA receptors in the spinal dorsal horn, thereby inducing spinal long‐term potentiation (LTP) at the C‐fiber synapses. This BDNF‐mediated LTP‐like state was identified as the cause of the blockade of spinal LTP induced by high‐frequency stimulation of the sciatic nerve in SNL rats. Ultimately, the authors confirmed that BDNF‐induced Src homology‐2 domain‐containing protein tyrosine phosphatase‐2 phosphorylation was necessary for the subsequent upregulation of GluN2B‐NMDA receptors, spinal LTP induction, and the development of abnormal pain [49]. Similarly, Li et al. demonstrated for the first time that Fyn kinase‐mediated phosphorylation of the tyrosine 1472 residue of GluN2B (pGluN2BY1472) is involved in BDNF‐induced spinal LTP and pain hypersensitivity and plays a crucial role in these processes. The BDNF‐Fyn‐GluN2B signaling cascade in the spinal dorsal horn may constitute a key mechanism underlying central sensitization and neuropathic pain development following peripheral nerve injury [50].

In studies related to chronic constriction injury (CCI) of the sciatic nerve, researchers have identified a significant time‐dependent increase in BDNF expression levels in the DRG of rats in the CCI model. Additionally, the overexpression of BDNF was found to increase the expression of purinergic ligand‐gated ion channel 7 receptor (P2X7 receptors) in the DRG, indicating that P2X7 receptors could be induced by BDNF [51]. Under CCI‐DRG conditions, BDNF activated the P2X7 receptor gene promoter, thereby promoting neuropathic pain. The microinjection of BDNF siRNA into the DRG blocked the high expression of BDNF in the CCI model and reduced the responses to mechanical, thermal, and cold stimuli [52]. Researchers found that exercise training could promote recovery in mice with sciatic nerve injury. The mechanism likely involves the enhancement of autophagy through the BDNF/AKT/mTOR signaling pathway, promoting the polarization of microglia in neuropathic pain, thereby alleviating pain. This study confirmed the efficacy of exercise training in alleviating neuropathic pain and proposed a new therapeutic target for neuropathic pain [53]. In addition to exercise training, studies investigating the potential mechanisms of electroacupuncture in treating neuropathic pain in CCI rats found that electroacupuncture could alleviate neuropathic pain by regulating the miR‐206‐3p/BDNF pathway and exerting anti‐inflammatory and anti‐apoptotic effects [54]. In recent years, significant progress has been made in the research of miRNAs as potential treatment targets in chronic pain, including research on the mechanism of neuropathic pain progression in rats with CCI of the sciatic nerve lacking miR‐30a‐3p, which some researchers believe is closely related to BDNF. miR‐30a‐3p targets the E‐calcium‐binding protein transcription activator (EP300), which upregulates BDNF at the epigenetic level by enhancing the acetylation of histones H3 and H4 in its promoter. The loss of miR‐30a‐3p enhances the colocalization of EP300 and BDNF in CCI rats, and the increase in EP300‐induced neuropathic pain by enhancing the level of neuronal BDNF. Therefore, the epigenetic modification of BDNF induced by miR‐30a‐3p and EP300 promoted neuropathic pain in CCI rats [55].

#### 2.1.2 The Mediating Role of BDNF in Reducing Neuropathic Pain

Studies have shown that prolonged high BDNF expression increases excitability in the spinal dorsal horn and mediates central sensitization, leading to synaptic changes. This may be one of the mechanisms underlying the development of neuropathic pain [56]. This specific mechanism may involve the activation of P2X4 purinergic receptors (P2X4Rs) in microglial cells by ATP in the spinal dorsal horn following peripheral nerve injury, leading to BDNF release. Through binding to the TrkB receptor, BDNF alters neuronal excitability and induces abnormal pain [57]. The effect of BDNF on the spinal cord has been observed in various neuropathic pain conditions, and its role in drug treatment for alleviating pathological pain is of significant importance.

In one study on HIV‐associated neuropathic pain pathology, researchers found that exposing sensory neurons to viral envelope proteins, such as the intrathecal injection of gp120, in an HIV pain model produced abnormal mechanical pain. This increased the expression of Wnt3a, β‐catenin, and BDNF in the mouse spinal cord. Blocking the Wnt or BDNF signaling pathways alleviates abnormal mechanical pain. Additionally, minocycline inhibits microglial activation, thereby reducing abnormal mechanical pain [58]. Furthermore, evidence suggests that 3,5,6‐trifluoro‐2′‐deoxyinosine (ICTF) can attenuate neuropathic pain induced by spinal nerve injury (SNI). ICTF achieves this by blocking the spinal BDNF/TrkB/KCC2 (the electroneutral K^+^‐Cl^−^ cotransporters) signaling pathway mediated by α7 nicotinic acetylcholine receptors (α7nachr), thereby inhibiting the activation of microglial cells and relieving SNI‐induced neuropathic pain. These findings indicate that ICTF may serve as an analgesic against neuropathic pain [59]. In addition to these drugs, researchers have found that fluorocitrate can reverse existing abnormal mechanical pain by inhibiting the upregulation of BDNF upregulation induced by activated astrocytes [60]. Simultaneously, curcumin, which plays a positive role in treating various neurological diseases, can exert therapeutic effects on neuropathic pain by downregulating the expression of the BDNF and Cox‐2 genes, in a manner mediated by p300/CREB‐binding protein histone acetyltransferase activity [61].

However, contrasting evidence has suggested that BDNF plays a protective role in the nervous system. Indeed, studies have indicated that exogenous BDNF treatment in neuropathic pain models can reduce pain‐associated behaviors [62]. For example, one study showed that hourly infusion of 1 μg BDNF significantly reduced mechanical hypersensitivity in the von Frey test in a rat model of neuropathic pain. Conversely, infusion of 20 μg/h BDNF enhanced the above test response. Conversely, the infusion of 0.5 and 10 μg/h BDNF did not affect mechanical hypersensitivity. Experimental data indicated that systemic BDNF treatment could selectively alter high‐threshold mechanical sensitivity, demonstrating the protective role of BDNF against injury‐induced hypersensitivity [63]. Similarly, researchers found that viral vector‐mediated BDNF overexpression alleviated chronic neuropathic pain in rats following spinal cord injury [64]. Furthermore, BDNF may play a crucial role in the antidepressant and anti‐injury effects of neuropathic pain. Antidepressants have been used to treat neuropathic pain and have shown beneficial effects in animal and clinical studies. Recent research in both animals and humans has suggested that antidepressants can increase central and plasma BDNF levels and that BDNF can mediate the therapeutic effects of antidepressants on neuropathic pain. This concept may aid the selection of specific types and optimal doses of antidepressants for the treatment of neuropathic pain [65].

### 2.2 BDNF and Inflammatory Pain

BDNF serves as the foundation for the development and maintenance of inflammatory pain in the spinal cord. During inflammation, immune cells accumulate in damaged areas, releasing pro‐inflammatory cytokines and NTFs. BDNF exerts neuroregulatory effects and promotes pain transmission through binding to the postsynaptic tyrosine kinase B (TrkB) receptor in the spinal dorsal horn. In a study by Lin et al., whether and how the BDNF–TrkB signal in the DRG participates in the process of inflammatory pain was investigated using the Complete Freund's Adjuvant (CFA) and TNF‐α induced rat hind paw inflammatory pain animal model. In conclusion, existing evidence indicates that inflammation and TNF‐α upregulate BDNF–TrkB signaling in the DRG. It has also been suggested that the upregulation of BDNF in the DRG, in addition to acting postsynaptically in the spinal dorsal horn, may also activate presynaptic TrkB receptors as autocrine and/or paracrine signals, regulating synaptic excitability in pain transmission, thereby promoting the occurrence of pain hypersensitivity [66]. P2RX4, which is primarily expressed in microglial cells and whose activation triggers the release of various pro‐inflammatory molecules is considered as one potential therapeutic target for chronic inflammatory pain. Previous studies have found that, under inflammatory conditions, P2RX4 in neurons can control the release of BDNF from peripheral sensory nerve terminals to the spinal dorsal horn, thereby mediating pain generation. Lalisse et al. discovered that in P2RX4‐deficient mice spinal dorsal horns, BDNF‐dependent signaling pathways, phosphorylation of Erk1/2 and the GluN1 subunit, as well as downregulation of the co‐transporter KCC2, which are triggered by peripheral inflammation, are all impaired. Ultimately, P2RX4 was shown to control the release of BDNF from sensory neurons, leading to heightened excitability in neurons during chronic inflammatory pain, while co‐localized with BDNF, showing upregulation during peripheral inflammation [67]. In studies on the mechanism by which TNF‐α upregulates BDNF levels, researchers have found that TNF‐α can activate astrocytes and microglial cells through stimulation. These activated glial cells may release the neurotrophic factor BDNF, leading to an increase in BDNF levels (PMID: 34877406). At the same time, activated microglial cells also produce pro‐inflammatory cytokines, which exacerbate inflammation.

In the rostral anterior cingulate cortex (rACC), BDNF is an activity‐dependent neuromodulator involved in the occurrence and persistence of inflammatory pain in the rACC. Wang et al. further conducted experiments using formalin‐induced inflammatory pain models in rats and found that BDNF activates mTOR to upregulate NR2B expression in the rACC, which is required for inflammatory pain‐related aversion [68]. Additionally, in one study on the association between BDNF and inflammatory pain in the rACC, some researchers found that BDNF was upregulated in the rACC and primary somatosensory cortex (S1) of rats with inflammatory pain. The authors thus concluded that BDNF‐dependent neuronal plasticity in the rACC is a key mechanism in the development and maintenance of chronic pain emotions [69].

Pro‐BDNF is an unprocessed product of the BDNF gene capable of binding to its receptor to exert biological functions. Studies have shown that in inflammatory pain, pro‐BDNF and its main p75 pan‐neurotrophin receptor are upregulated in nerve fibers and local inflammatory cells. Multiple studies have shown that its upregulation exacerbates inflammation. The administration of monoclonal anti‐pro‐BDNF antibodies can alleviate various types of inflammatory and surgical pain. Their experiments demonstrated that pro‐BDNF is a potential pain mediator, and that pretreatment with anti‐pro‐BDNF can mitigate the development of inflammatory pain [70]. Furthermore, Li et al. demonstrated that activated pro‐BDNF/p75NTR signaling in the spinal cord is involved in CFA‐induced inflammatory pain development [71].

### 2.3 Mechanisms of BDNF Therapy for Mental Disorders

Previous studies have indicated that BDNF in the brain is associated with the development of mental disorders [7]. The mechanisms underlying the role of BDNF therapy in mental disorders are outlined below. BDNF serves as a crucial source of the driver of various neurotransmitters, including dopamine, serotonin, norepinephrine, and gamma‐aminobutyric acid (GABA) in the CNS, which together play a role in regulating synaptic plasticity. In animal models of Parkinson's disease (PD), treatment with BDNF enhances the survival of dopaminergic neurons, improving dopaminergic neurotransmission and motor performance [72]. BDNF and the PI3k/Akt signaling pathway are involved in neuroregeneration and anti‐apoptotic activity. Research has confirmed that the natural polyphenol curcumin can exert positive neuroprotective effects in PD by activating the BDNF and PI3k/Akt signaling pathways [73]. Depression and cognitive impairment are common nonmotor symptoms of PD. Studies have shown that BDNF may be involved in the pathological processes underlying depression in PD patients, thereby playing a significant role in cognitive function and depression [74]. Additionally, research suggests that in Alzheimer's disease (AD), BDNF acts by inhibiting neuroinflammation and glial cell activity [75]. Beta‐amyloid (Aβ) is considered a major cause of synaptic dysfunction and cognitive impairment in AD. Several studies have proposed that Aβ primarily reduces BDNF, affecting human cognitive function by lowering CREB phosphorylation [76]. Moreover, growing evidence suggests a positive correlation between moderate‐intensity aerobic exercise, cognitive function, and memory in the treatment of AD, with BDNF as a potential key mechanism. This involves exercise‐driven synthesis and the accumulation of neuroactive metabolites (such as myokines and ketones) in the periphery and hippocampus, which enhance BDNF expression. Following voluntary exercise, both BDNF mRNA and protein levels significantly increased in rodents. Similarly, BDNF generated through aerobic exercise and cardiovascular health have been shown to be positively correlated with human hippocampal volume and function. As such, the connection between BDNF, exercise, and cognition holds crucial therapeutic significance for preventing and improving memory loss and cognitive impairment in AD and related dementias [77]. In autism spectrum disorders, BDNF may exert anti‐autism effects by modulating the interaction between glutamate receptors (GluR) and GABA receptors (GAD) [78]. Additionally, the signaling pathways mediated by BDNF and its receptor TrkB play key roles in the pathophysiology of depression and the therapeutic mechanisms of antidepressant drugs [79]. Moreover, research has indicated that plasma BDNF levels are decreased in patients with severe depression, whereas postmortem hippocampal tissues from patients taking antidepressant drugs show increased BDNF levels. In this context, BDNF may regulate the function of PSD protein and PSD‐95/PSD‐97 complexes by binding to density proteins, contributing to their neuroprotective mechanisms [80].

### 2.4 Future Directions in BDNF Therapy for Mental Disorders

BDNF is a NTF that plays a variety of roles in the adult CNS. There is substantial evidence to indicate that a significant reduction in BDNF levels is associated with mood disorders. Further, BDNF plays a crucial role in most antidepressant treatments and is further associated with neurodegenerative diseases and neurological disorders such as schizophrenia [81]. Considerable research has been conducted to evaluate the therapeutic potential of BDNF in various brain disorders, yielding promising results, although these studies have several limitations. Despite this, progress has been made in addressing this issue through various methods, such as gene therapy [82] and carrier‐free stabilized nanocapsules [83], allowing the intranasal administration of BDNF nanoformulations [84]. Other approaches include the use of TrkB receptor ligands, small‐molecule BDNF mimetics, and functional antibodies [85], as well as compounds that enhance BDNF synthesis, delivery, and signal transduction, including natural products [86, 87]. The therapeutic potential of BDNF has also sparked significant interest [88]; however, these collectively termed direct and indirect approaches based on BDNF therapy still face many challenges. Further research is required to elucidate the network‐specific functions of BDNF, as well as to explain how it regulates cognition and behavior. Other factors to consider include systemic side effects, interactions with other diseases that may result from elevated BDNF levels, and the potential impact of different polymorphisms on BDNF‐based therapies.

In summary, the BDNF‐mediated TrkB signaling pathway controls various neuronal functions and is involved in multiple molecular mechanisms that counteract various pathophysiological processes crucial to neurological and psychiatric disorders. These processes include synaptic regeneration and the maintenance of synaptic activity and structure, immune regulation against excessive astrocyte activity, abnormal production of inflammatory mediators, and secondary neural regulation altering dopamine and serotonin transmission. Overall, BDNF‐based therapeutic approaches hold significant potential for treating various neurological and psychiatric disorders. However, a series of challenges remain to be addressed.

## 3 BDNF's Impact and Mechanisms in Cancer

### 3.1 Role of BDNF and TrkB in the Pathogenesis of Cancer

Under physiological conditions, BDNF and TrkB are widely distributed in both the central and peripheral tissues. However, despite maintaining normal distribution under cancer conditions, BDNF/TrkB expression are often upregulated. Research has found that the upregulated activation signals of BDNF/TrkB in tumor cells stimulate a series of downstream pathways, including PI3K/AKT, Ras–Raf–MEK–ERK, PLC‐γ, as well as the transactivation of epidermal growth factor receptor. The activation of these pathways induces oncogenesis by promoting cancer cell growth, proliferation, survival, migration, and EMT, while concurrently reducing tumor regression, recurrence, and chemotherapy sensitivity [37].

Some studies have further shown that BDNF/TrkB can increase neuroblastoma metastasis via the PI3K/Akt/mTOR and MAPK pathways, indicating that BDNF/TrkB and its downstream targets may be potential therapeutic targets for treating neuroblastoma metastasis [42]. Meanwhile, in one study on the molecular mechanisms involved in the progression of prostate cancer, both BDNF and TrkB were found to be overexpressed in prostate cancer tissues and could promote tumor growth. The elevated expression of TrkB has also been closely related to lymph node metastasis and advanced prostate cancer, indicating that the BDNF/TrkB pathway is crucial for the progression of prostate cancer. This may provide a new therapeutic strategy for the treatment of advanced prostate cancer [89].

In addition to these two cancers, this upregulation has been observed in various other tumors, including lung cancer (small cell and non‐small cell) [90], multiple myeloma [91], hepatocellular carcinoma [92], ovarian cancer [93], pancreatic ductal adenocarcinoma [94], glioblastoma [95], head and neck squamous cell carcinoma, breast cancer [96], gastric cancer [97], colorectal cancer, gallbladder cancer, and cervical cancer [89].

### 3.2 Signaling Pathways of BDNF and TrkB in the Pathogenesis of Cancer

**FIGURE 3:**
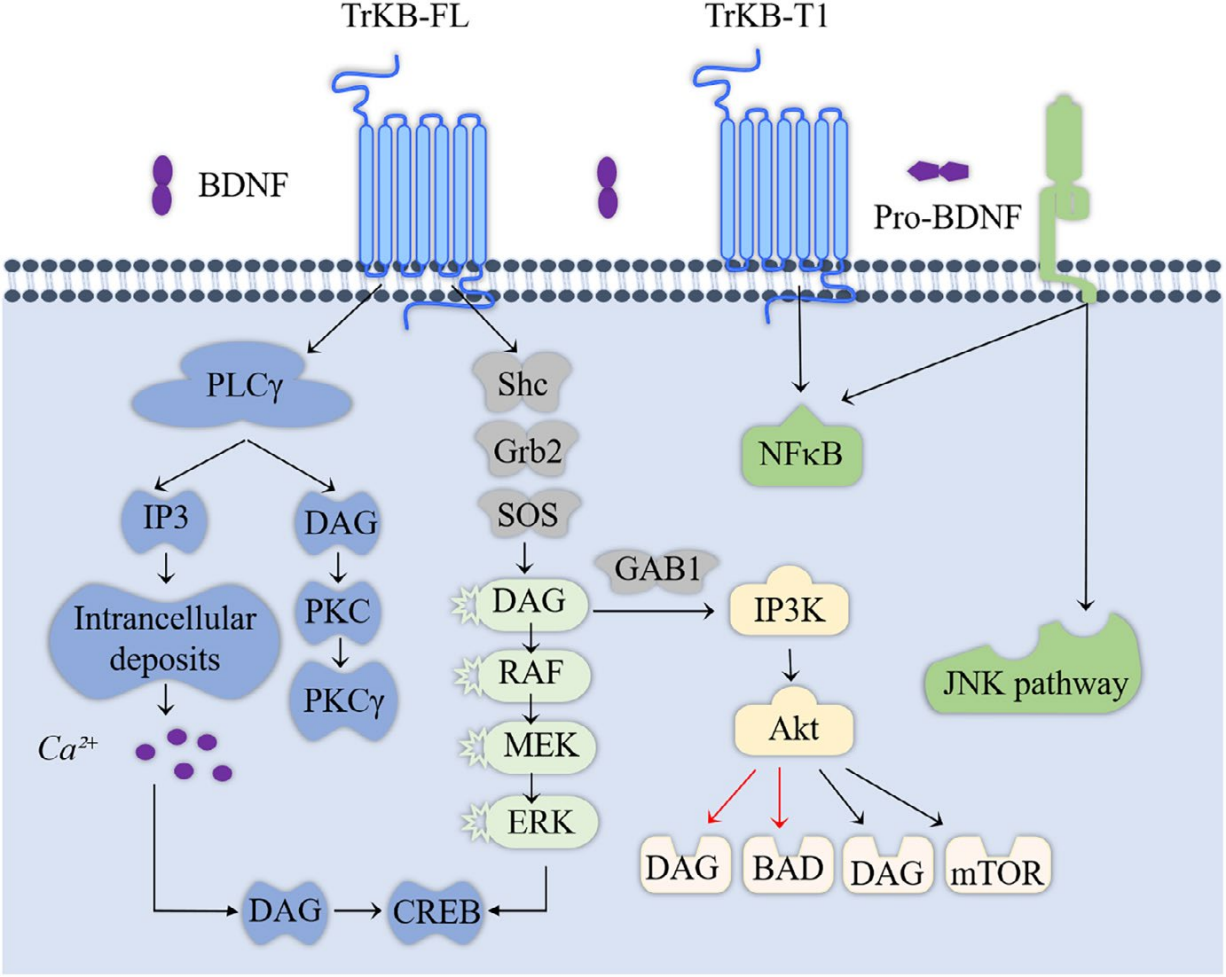
Involvement of BDNF–TrkB in mediating cancer‐promoting pathways. The phospholipase C‐γ (PLC‐γ) pathway activates inositol trisphosphate (IP3) receptors, promoting the release of intracellular Ca^2+^. Elevated intracellular calcium levels increase CaMK activity, leading to increased synaptic plasticity in neurons. Furthermore, PLC‐γ allows for the generation of diacylglycerol (DAG). Calcium release and DAG formation indirectly regulate a myriad of cellular activities through the PI3K and MAPK pathways and the direct activation of protein kinase C (PKC). Additionally, TrkB receptor phosphorylation of PLC‐γ drives another pathway in the regulation of transcription factors such as cAMP response element‐binding protein (CREB). This axis promotes vascular endothelial VEGF expression and angiogenesis.

BDNF promotes cancer progression, reduces chemotherapy responsiveness, and enhances angiogenesis by increasing cancer cell survival, proliferation, migration, and invasion. Figure 3 illustrates the pathways through which BDNF–TrkB mediates cancer‐related effects [39, 98]. TrkB belongs to the neurotrophic tyrosine kinase receptor (NTRK2) family, encoded by the NTRK2 gene, and interacts with other ligands such as neurotrophin‐3 (NT3) and neurotrophin‐4 (NT4) [4, 99]. Research has indicated that the dose‐ and time‐dependent activity of TrkB is associated with the functional BDNF axis [100]. Binding of BDNF to TrkB induces receptor dimerization, which leads to the autophosphorylation of the RTK domain. This activation initiates intracellular signaling pathways [101] that mediate the functions of BDNF.

#### 3.2.1 MAPK Signaling Pathway

The upstream regulatory factor of the MAPK signaling pathway is MAPK, and each MAPK signaling cascade includes at least three layers: MAP3K, MAPKK, and MAPK [102]. Research has suggested that the c‐Jun N‐terminal kinase (JNK) and p38 MAPK pathways are primarily associated with stress and apoptosis, while the ERK/MAPK signaling pathway is linked to cell proliferation and differentiation, in which it plays a crucial role [103]. Therefore, activating the MAPK signaling pathway can promote cell proliferation, inhibit apoptosis, and stimulate angiogenesis, thereby influencing tumor progression [104]. MAPK activity is regulated by ERK1/2 or transcriptional activation factor‐1 (MAP‐1) [105]. Multiple studies have indicated that elevated levels of ERK1/2 phosphorylation are associated with shortened survival times for patients with cancer in human breast [106], pancreatic [107], colorectal [108], and liver cancer [109] tissues. Excessive activation of the MAPK signaling pathway is considered a hallmark of carcinogenesis in many tumors, leading to cancer cell metastasis [110].

#### 3.2.2 PI3K/Akt/mTOR Pathway

The PI3K/Akt/mTOR signaling pathway is a major cellular signaling pathway that plays a crucial role in maintaining fundamental cellular functions, contributing significantly to the occurrence, development, and metastasis of tumors. The PI3K/Akt/mTOR pathway regulates cell proliferation, growth, size, metabolism, and movement. The constituent genes of this pathway have been extensively studied and found to be universally activated in human cancers, where they function to promote cancer progression [111]. Numerous studies have also investigated their roles in cancer. For example, Durán‐Maldonado et al. conducted experiments and found that activation of the PI3K/Akt pathway is associated with the occurrence and metastasis of melanoma in mouse melanoma cell lines [112]. Similarly, in human ovarian cancer, the activation of the PI3K/Akt signaling pathway is linked to increased growth and metastasis of tumor cells. Therefore, inhibition of the PI3K/Akt signaling pathway may represent a potential approach for treating ovarian cancer [113]. In addition, research has shown that the binding of BDNF to TrkB can activate PI3K, MAPK, and PLC‐γ pathways [114, 115, 116]. Akt and mTOR are two downstream targets in the PI3K pathway [42]. Therefore, BDNF/TrkB and its downstream targets are potential targets for cancer therapy.

#### 3.2.3 PLC‐γ Pathway

The PLC‐γ pathway activates inositol 1,4,5‐triphosphate (IP3) receptors, resulting in the release of intracellular Ca^2+^. Elevated intracellular calcium levels increase CaMK activity, thereby contributing to enhanced synaptic plasticity in neurons [117]. Additionally, PLC‐γ generates diacylglycerol (DAG). Calcium release and DAG formation regulate extensive cellular activity through indirect activation of the PI3K and MAPK pathways and direct activation of protein kinase C [118]. Simultaneously, phosphorylation of the TrkB receptor by PLC‐γ drives an alternative pathway that ultimately influences transcription factors such as CREB [119].

### 3.3 BDNF as a Biomarker for Cancer Diagnosis and Prognosis

Patients with cancer are commonly diagnosed only at an advanced stage, contributing to a poor prognosis. Therefore, identifying new biomarkers for diagnosis and prognosis can enhance treatment strategies and improve overall cancer outcomes [120]. Several studies have explored the potential of BDNF, its receptors, and signaling pathways as biomarkers for various cancers. In a female breast cancer patient cohort, Patani, Jiang, and Mokbel concluded that higher BDNF expression was significantly associated with poorer survival rates and adverse prognosis. They further suggested that BDNF could serve as a prognostic marker for early cancer detection and improve treatment outcomes [121]. Another study proposed that BDNF antisense RNA (BDNF‐AS) may be a prognostic biomarker of retinoblastoma, as low expression of BDNF‐AS is correlated with advanced clinical staging and shorter overall survival, and overexpression of BDNF‐AS in retinoblastoma cells inhibits cancer cell migration and proliferation [120]. Moraes et al. investigated the role of the BDNF/TrkB/Akt pathway in oral squamous cell carcinoma (OSCC), finding elevated activity in malignant cells. BDNF and Akt have been suggested as potential prognostic biomarkers for patients with early‐stage OSCC [122]. Numerous studies have identified BDNF and its receptors as diagnostic markers for gastric cancer [123], small cell lung cancer [124], and pancreatic cancer [125]. Virtually all of these studies affirm the potential of BDNF‐related indicators in cancer diagnosis and prognosis.

### 3.4 BDNF Receptors and Associated Signaling Pathways as Therapeutic Targets for Cancer

In addition to serving as a diagnostic and prognostic biomarker, BDNF may affect cancer treatment through various mechanisms. For example, BDNF plays a role in regulating the cell cycle, cell proliferation, and apoptosis and may potentially inhibit the self‐renewal of cancer cells. In one study of melanoma in mice, researchers observed BDNF‐mediated apoptosis and demonstrated that this process could inhibit melanoma cell self‐renewal by inducing cell cycle arrest and suppressing cell proliferation. Additionally, researchers discovered a BDNF signaling pathway that inhibits breast cancer by regulating specific kinases and cytokines [126].

Given the significance of BDNF in cancer treatment, research into its targets has begun to evolve. One such target is BDNF receptor 2, which plays a crucial role in apoptosis. Recent studies have indicated that BDNF receptor 2 regulates Bcl‐xL by interacting with the Bcl‐2 family member BAX [127]. Studies have further identified BAX as one of the most critical regulatory factors in the BDNF signaling pathway, promoting BAX protein expression and the generation of apoptosis‐related proteins Bax and Bcl‐xL in breast cancer cell lines [128]. However, research on BDNF receptors and their associated signaling as therapeutic targets for cancer is still in its early stages, and more contributions from researchers in this field are anticipated.

## 4 Impact and Mechanisms of Action of BDNF in Cardiovascular Diseases

### 4.1 Association Between Depression and Cardiovascular Diseases

The findings of many studies have indicated that depression is associated with an increased risk of all‐cause and cardiovascular disease mortality among adults in China [129]. In the context of depression, which is a common comorbidity of coronary heart disease (CHD), Tschorn et al. analyzed the relationship between BDNF and depression in a sample of CHD patients, finding that individuals with depression typically exhibited decreased BDNF concentrations, and that serum BDNF levels were correlated with cardiovascular dysfunction. Based on these results, they proposed that when studying the relationship between depression and BDNF, comorbidities and physical conditions [130]. Studies have suggested that in healthy individuals, BDNF levels initially increase with age, subsequently remaining stable after adulthood. However, BDNF levels decrease in individuals with cardiovascular diseases or chronic mental disorders. Further, plasma BDNF levels in patients with depression are significantly lower than those in individuals with normal BDNF levels, and this situation is more severe in older individuals [131]. The occurrence of depression is causally related to cardiovascular diseases because lower BDNF levels are associated with various cardiovascular risk factors and metabolic syndromes [132].

### 4.2 Potential Role of BDNF in Cardiovascular Diseases

Numerous studies have indicated a significant role for BDNF in cardiovascular diseases. Experimental results have indicated that BDNF loss contributes to chronic ischemic heart failure and that TrkB agonists can improve ischemic left ventricular dysfunction by supplementing myocardial BDNF. Another BDNF‐based approach to preventing chronic ischemic heart failure involves the direct stimulation of cardiac β3AR or β‐blockers (by upregulating β3AR) [133]. Manni et al. found that plasma BDNF levels were generally reduced in patients with acute coronary syndrome, suggesting a potential protective role for BDNF [134]. Furthermore, various risk factors for cardiovascular diseases have been identified as independent determinants of low plasma BDNF levels in patients with angina, and reduced plasma BDNF may be associated with future coronary events and mortality [135]. BDNF variants also play a role in cardiovascular diseases. For example, in one experiment by Sandrini et al., the BDNF Val66Met polymorphism was linked to adverse cardiac remodeling after myocardial infarction and influenced macrophage phenotypes in both humans and mice. These findings suggest a novel cellular mechanism by which BDNF gene variations may affect cardiovascular diseases [136].

### 4.3 Mechanisms of BDNF–TrkB Expression in Cardioprotection

BDNF exerts a cardioprotective function by promoting the proliferation of myocardial cells and shielding them from ischemic and hypoxic damage. However, the specific mechanisms remain unclear [133]. Research has indicated that TrkB expression in myocardial cells decreases after cardiac ischemia and that the binding of BDNF to p75NTR also diminishes [40]. Under hypoxic conditions, BDNF activates p75NTR and converts it into the TrkB receptor, thereby promoting the proliferation of myocardial cells. Reactivation of p75NTR after hypoxia enhances BDNF activity. Therefore, upregulation of BDNF expression under hypoxic conditions may be achieved through the activation of p75NTR [137]. Thus, BDNF likely protects the heart by inhibiting apoptosis. In ischemia/reperfusion injury, the expression of BDNF in myocardial cells is significantly decreased, and the expression of proteins associated with apoptosis, such as caspase‐3 and cleaved‐caspase‐9, is also markedly decreased in myocardial cells. Thus, BDNF expression in myocardial cells can reduce apoptosis by suppressing the expression of caspase‐3 and cleaved‐caspase‐9 [138].

Additionally, some researchers have experimentally shown that BDNF can enhance normal cardiomyocyte Ca^2+^ cycling, contraction, and relaxation through Ca(2+)/calmodulin‐dependent protein kinase II (CaMKII), thereby exerting a protective effect on the heart [139]. Meanwhile, several studies have further found that in cultured hippocampal neurons, BDNF–TrkB can induce calcium influx by activating its downstream PLC‐γ‐IP3 signaling pathway, leading to an increase in intracellular calcium levels. This elevation in calcium level facilitates the activation of Myosin II, which promotes the translocation of Drp1 from the cytoplasm to the mitochondria, thereby accelerating mitochondrial fission [140]. In addition, research has found that when BDNF binds to its receptor TrkB in the brain, it activates the PI3K and Akt kinases. Akt subsequently activates mTOR, which stimulates the translation of mRNAs encoding the neuronal glucose transporter GLUT3 and monocarboxylate transporter 2, thereby enhancing the uptake of glucose and lactate by cells [141]. Based on these results, we speculate that a similar mechanism may exist in cardiomyocytes, where BDNF–TrkB, by activating its downstream pathways, promotes mitochondrial fission and ATP production, thereby enhancing the energy supply to cardiomyocytes and exerting a protective effect on the heart.

Examination of the mechanisms by which BDNF promotes heart function has indicated that the BDNF–TrkB signaling pathway likely participates in cardioprotective mechanisms following myocardial ischemia/reperfusion injury. However, because of the complexity of cardiac function recovery after myocardial infarction, determining whether these factors act independently is challenging.

## 5 Conclusion

BDNF plays a crucial role in several human diseases. Numerous clinical and animal experimental studies have demonstrated that BDNF alleviates neuronal damage and death in various diseases, and its protective effects on the cardiovascular system have been confirmed. BDNF is associated with the occurrence and development of various cancers as well as several cardiovascular, neurodegenerative, and neurological diseases. In particular, the BDNF/TrkB signaling pathway plays a key role in maintaining the normal function of neuronal cells in the nervous system, and its abnormalities are directly linked to various neurodegenerative and neurological diseases. The BDNF/TrkB signaling pathway is closely linked to the development of cancer and cardiovascular diseases. Based on these findings, the potential application of BDNF as a biomarker for the diagnosis, prognosis, and treatment of cancer and cardiovascular diseases has been validated. However, there is still room for further research and contributions, especially in the exploration of treatment targets and methods, particularly for diseases such as cancer. Continued efforts from researchers are required to address the existing gaps and advance our understanding of these areas.

## Author Contributions

Wu Liu, Qiwen Liu, Min Lei, and Jinxia Nie wrote the main manuscript text. Rongyi Huang, Yan Mei, Dan Pan, and Yong Chen prepared Figures 1, 2, 3. All authors reviewed the manuscript.

## Ethics Statement

The authors have nothing to report.

## Consent

The authors have nothing to report.

## Conflicts of Interest

The authors declare no conflicts of interest.

## Data Availability

Research data are not shared.
